# Pharmacological treatment and psychiatric polypharmacy in forensic psychiatric care in Sweden

**DOI:** 10.1192/j.eurpsy.2025.10031

**Published:** 2025-06-11

**Authors:** Taalke Maria Sitter, Suvi Virtanen, Hanna Edberg, Peter Andiné, Anja Fernqvist, Ebba Noland, Tatja Hirvikoski, Thomas Nilsson, Zheng Chang

**Affiliations:** 1 Department of Medical Epidemiology and Biostatistics, Karolinska Institutet, Stockholm, Sweden; 2 School of Educational Sciences and Psychology, University of Eastern Finland, Joensuu, Finland; 3 Swedish Prison and Probation Services, Norrköping, Sweden; 4 Northern Stockholm Psychiatric Clinic, Region Stockholm, Stockholm, Sweden; 5 Department of Psychiatry and Neurochemistry, Centre for Ethics, Law and Mental Health (CELAM), Institute of Neuroscience and Physiology, Sahlgrenska Academy, University of Gothenburg, Gothenburg, Sweden; 6 Forensic Psychiatry, Sahlgrenska University Hospital, Gothenburg, Sweden; 7 Department of Forensic Psychiatry, National Board of Forensic Medicine, Gothenburg, Sweden; 8 Department of Social Work, Umeå University, Umeå, Sweden; 9 Sundsvall Forensic Psychiatric Centre, Region Västernorrland, Sundsvall, Sweden; 10 Centre for Psychiatry Research, Region Stockholm, Stockholm, Sweden; 11 Habilitation & Health, Stockholm County Council, Stockholm, Sweden; 12 Department of Women’s and Children’s Health, Center for Neurodevelopmental Disorders at Karolinska Institutet (KIND), Karolinska Institutet, Stockholm, Sweden

**Keywords:** antipsychotics, forensic psychiatric care, pharmacological treatment, coercive measures, recidivism

## Abstract

**Background:**

Patients in forensic psychiatric care (FPC) are commonly treated with a wide range of psychotropic medications. There is, however, a lack of understanding regarding how pharmacological treatment and psychotropic polypharmacy are used throughout care.

**Method:**

This register-based cohort study included patients admitted to FPC in Sweden between 2009 and 2020. We estimated the prevalence of the use of major psychotropic medication, as well as psychotropic polypharmacy, at admission and discharge. We also examined the change in antipsychotic use after admission.

**Results:**

In total, 1962 patients were included. Antipsychotics were the most used psychotropic medication class, with 86.2% (95%CI: 84.5–87.8) of patients receiving at least one typical or atypical antipsychotic at admission. Changes in the antipsychotic regime were more common at the beginning of FPC, compared to later time points. Within the subgroup of patients discharged during the study period (n = 561), there was a reduction in the use of typical antipsychotics (admission: 34.9%; discharge: 26.6%) and hypnotics and sedatives (admission: 37.4%; discharge: 28.1%). Other major medication classes remained relatively stable. The prevalence of psychiatric polypharmacy at admission was 70.6% (95%CI: 68.5–72.7) and remained similar during care.

**Conclusions:**

Our study documented a high prevalence of antipsychotic use and psychotropic polypharmacy through FPC. Further, a high level of off-label antipsychotic use and antipsychotic polypharmacy was observed. Stronger evidence regarding the effectiveness and safety of these treatment strategies is needed.

## Introduction

In most high-income countries around the world, criminal offenders with severe mental disorders are treated based on special regulations in the legal system, which can lead to forensic psychiatric care (FPC) instead of prison. FPC differs from regular psychiatric care, as it is in many countries associated with the criminal justice system and has a patient group with severe psychiatric disorders, of which a majority have committed violent crimes. Further, in addition to reducing mental health problems, it aims to reduce the risk of criminal recidivism [1, 2]and is often characterized by extended care durations [3]. In Sweden, the number of patients in forensic psychiatric care has increased over the last few years, with currently more than 2000 patients in care [4]. This trend is also observed in other European Countries, including the Netherlands, the United Kingdom, and Austria [5, 6].

In the Swedish FPC system, most patients have some form of psychotic, neurodevelopmental, or substance use disorder [4]. A wide range of psychotropic medications are frequently used in this setting, both in monotherapy and polypharmacy [7–9]. Psychotropic polypharmacy is considered to be the simultaneous prescription of two or more psychotropic medications [10]. In 2018, it was reported that a majority of patients in FPC in Sweden received antipsychotics, with a high prevalence of antipsychotic polypharmacy – the simultaneous prescription of two or more different antipsychotics [7]. Those assessments were, however, cross-sectional. Currently, there is a lack of understanding of when and how antipsychotics and other psychotropic medications are used during FPC and how drug regimens change during the care process.

To generate robust evidence and better support clinicians in forensic psychiatric clinics, it is essential to expand our understanding of current pharmacological treatment practices in FPC. Due to the above-mentioned differences, descriptions of pharmacotherapy from general psychiatric care might have only limited comparability [11–13]. The presented study aims to describe the longitudinal pattern of pharmacological treatment in FPC and to estimate the prevalence of major psychotropic medication classes and polypharmacy during care, taking comorbidities and time in care into consideration.

## Methods

### Study design and data source

This study is a register-based cohort study utilizing data from the Swedish National Forensic Psychiatric Register (SNFPR/RättspsyK), which, to our knowledge, is the only nationwide forensic psychiatric patient register worldwide. SNFPR has a high degree of coverage, capturing 84–96% of patients since 2009, with 25 of the country’s 26 forensic psychiatry units reporting to it today [14–16]. SNFPR includes records on new registrations, yearly follow-up, transfers between clinics, and discharge or death.

In Sweden, a person who commits a crime can be sentenced to FPC instead of prison, if the court and a forensic psychiatric assessment conclude that the convicted person has a severe mental disorder [17]. While “severe mental disorder” in Swedish legislation is considered a medicolegal term, rather than strictly medical, both the type and degree of the mental disorder are usually considered critical factors.

### Study population

All patients registered into SNFPR and starting FPC between 01/01/2009 and 01/01/2020 were included in the study. Individuals sentenced to FPC more than once during the study period were included only with their first sentence after 2009. In Sweden, the median duration of FPC is 7.5 years, including inpatient and outpatient care [18]. Therefore, the available follow-up period of 11 years does not cover the entire sentence for all individuals. Only including patients with both admission and discharge within the study period would introduce selection bias, as patients with shorter stays would be overrepresented. Therefore, all patients admitted to FPC between 2009 and 2020 were included, independently from their discharge status in 2020. Subgroups were created for different aspects of the analysis: (1) the entire sample, (2) subgroups based on discharge status at the end of the study period, and (3) patients registered before 01/01/2016 (eFigure 1 in Supplementary Material), allowing at least 4 years of follow-up. This study was approved by the Swedish Ethical Review Authority (reference number 2022-07201-02).

### Measurements

The primary outcome of interest was psychotropic medication use during FPC. Information on standing prescriptions and pro re nata medications that have been given on more than three occasions in the prior week is recorded at every data record. We examined major classes of psychotropic medications: (typical and atypical) antipsychotics (ATC: N05A, except N05AN01; eTable 1), antidepressants (N06A), attention deficit/hyperactivity disorder (ADHD) medication (N06B), hypnotics and sedatives (N05B and N05C), antiepileptic drugs (N03A, except N03AG01 and N03AF01), opioids (N02A, except N02AE01), drugs used in addictive disorders (N07B and N02AE01), and mood stabilizers (N05AN01; N03AG01; N03AF01). The second outcome was psychotropic polypharmacy (use of ≥2 psychotropic medications, including same-class polypharmacy) [10]. The third outcome was the antipsychotic use pattern, based on the type and number of antipsychotics used. Mutually exclusive groups were: (1) use of no antipsychotics, (2) one typical antipsychotic, (3) more than one typical antipsychotic, (4) one atypical antipsychotic, (5) more than one atypical antipsychotic, and (6) both typical and atypical antipsychotics. Missing medication information, assessment, and treatment are described in eMethod 1 in the Supplementary Material. Follow-up time was measured from admission until discharge or last follow-up. To describe medication changes throughout care, the number of data records was used as a standardized method to define time points. A description of how data records were handled in the analysis can be found in eMethod 2.

Up to three ICD-10 diagnoses were recorded in every data record of a patient. Comorbidities of interest include psychiatric disorders, which are not characterized by an episodic nature and can be expected to influence treatment decisions throughout FPC: Schizophrenia Spectrum Disorder (SSD) (F20-F29), Substance Use Disorder (SUD) (F1_.2; F1_.7), Bipolar Disorder (F31), Recurrent Depressive Disorder (F33), ADHD (F90), Personality Disorder (F60-F69), Autism (F84.0, F84.1, F84.5, F84.8, and F84.9), and Intellectual Disability (F70-F79). All diagnoses were treated equally, and the order of diagnoses was not considered.

### Analysis

We described the demographic and psychiatric characteristics of the cohort. Further, we estimated the prevalence of the use of major classes of psychotropic medication and psychotropic polypharmacy in the total sample and separated by discharge status. We assessed differences between admission and discharge in the group of patients discharged before 2020, with a McNemar’s test for paired nominal data. Additionally, we tested medication use differences between subgroups of discharge status and sex at admission with a chi-squared test. Adjustments due to multiple testing are described in eMethod 3. Further, we estimated the prevalence of medication use throughout the first four follow-ups after admission and presented them graphically by psychiatric diagnoses. Lastly, to describe changes between treatment regimens within the first 4 years of treatment, we created Sankey diagrams on the type and number of antipsychotics used. For this part of the analysis, only patients admitted to care before 01/01/2016 were included in order to follow the first four follow-ups after admission. We created two additional Sankey diagrams to represent the change between admission and discharge and between admission and the fourth follow-up. The package “networkD3” in R 4.3.1 was used to generate the Sankey diagrams, while the remaining data analysis was performed using STATA 18.

## Results

In total, 1962 patients fulfilled the inclusion criteria. Of those, 677 had their entire treatment documented, with 619 being discharged before the end of the study period, while 58 had died during care (eFigure 1). Characteristics of the sample and the subgroups are presented in Table 1. In the full cohort, 59.7% of patients received two or more psychiatric diagnoses during FPC. The most common diagnosis was SSD (74.4%), followed by SUD (31.2%) and personality disorder (24.5%) (Table 1). Common comorbidities include SSD with SUD (25.1%) and SSD with personality disorder (14.8%) (Table 2 and eFigure 2).

**Table 1.** Characteristics of the study sample

	Total sample	Not discharged before 2020	Discharged before 2020	Died during FPC
Total n	1962	1285	619	58
Sex n (%)				
Male	1614 (82.3)	1072 (83.4)	492 (79.5)	50 (86.2)
Female	348 (17.7)	213 (16.6)	127 (20.5)	8 (13.8)
Median age at admission in years	33.6	32.4	36.9	40.7
Sentence n (%)				
FPC with SCS^a^	1482 (75.5)	1066 (83.0)	371 (59.9)	45 (77.6)
FPC without SCS^a^	480 (24.5)	219 (17.0)	248 (30.1)	13 (22.4)
Median follow-up time in years (IQR)	3.7 (1.9–6.0)	4.1 (2.1–6.7)	2.9 (1.7–4.8)	3.0 (1.6–6.0)
Place of birth n (%)				
Sweden	1273 (64.9)	833 (64.8)	394 (63.6)	46 (79.3)
Other European country	206 (10.5)	126 (9.8)	79 (12.3)	<5 (−)
Non-European country	483 (24.6)	326 (25.4)	149 (24.1)	8 (13.8)
Psychiatric Diagnosis n (%)				
Schizophrenia Spectrum disorder	1460 (74.4)	990 (77.0)	428 (69.1)	42 (72.4)
Substance use disorder	613 (31.2)	429 (33.4)	156 (25.2)	28 (48.3)
Bipolar disorder	165 (8.4)	90 (7.0)	68 (11.0)	7 (12.1)
Recurrent depressive disorder	44 (2.2)	22 (1.7)	20 (3.2)	<5 (−)
ADHD^b^	258 (13.2)	182 (14.2)	66 (10.7)	10 (17.2)
Personality disorder	481 (24.5)	329 (25.6)	132 (21.3)	20 (34.5)
Autism	363 (18.5)	253 (19.7)	99 (16.0)	11 (19.0)
Intellectual disability	278 (14.2)	202 (15.7)	70 (11.3)	6 (10.3)

a Special court supervision.

b Attention-deficit/hyperactivity disorder.

**Table 2.** Prevalence of psychiatric diagnosis in patients with and without schizophrenia spectrum disorder

Other long-term psychiatric diagnosis^a^	Patients with Schizophrenia spectrum disorder N (%)	Patients without Schizophrenia spectrum disorder N (%)
Substance use disorder	493 (25.1)	120 (6.1)
Bipolar disorder	57 (2.9)	108 (5.5)
Recurrent depressive disorder	11 (0.6)	33 (1.7)
ADHD	160 (8.2)	98 (5.0)
Personality disorder	290 (14.8)	191 (9.7)
Autism	166 (8.5)	197 (10.0)
Intellectual disability	154 (7.9)	124 (6.2)
No other chronic psychiatric disorder	578 (29.5)	40 (2.0)

a Diagnoses (including both primary and secondary), which are not characterized by an episodic nature and can be expected to influence treatment decisions throughout FPC.

### Prevalence of medication use

Antipsychotics were the most used psychotropic medication class, with 86.2% (95%CI: 84.5–87.8) of patients receiving at least one antipsychotic at admission. Atypical antipsychotics (62.5%, 95%CI: 60.2–64.7) were used more frequently than typical ones (42.7%, 95%CI: 40.4–45.0). Other frequently used psychotropic medications at admission include hypnotics and sedatives (37.9%; 95%CI: 35.6–40.1) and antidepressants (32.2%, 95%CI: 30.0–34.4) (Table 3). Antiepileptics were used by more female (12.5%; 95%CI: 9.1–16.5) than male patients (5.5%; 95%CI: 4.4–6.8) at admission (p < 0.001), while the prevalence of use of other medication classes showed no significant sex differences (eTable 2A). The group of patients without discharge before 2020, compared to those discharged, had higher rates of typical antipsychotic use at admission (45.9% versus 34.9%, p < 0.001), which is also visible in higher general antipsychotic use numbers (88.6% versus 81.6%; p < 0.001) (eTable 2B).

**Table 3.** Use of psychotropic medications at admission and discharge

Time point	At admission						At discharge		Comparison admission and discharge^a^
	Total sample (n = 1834)		Individuais not discharged before 2020 (n = 1216)		Individuals discharged before 2020 (n = 561)		Individuals discharged before 2020 (n = 531)		
Sample (patients with non-missing medication information)	N	% (95% CI)	N	% (95% CI)	N	% (95% CI)	N	% (95% CI)	p-value
Antipsychotics	1581	86.2 (84.5–87.8)	1072	88.6 (86.2–89.9)	458	81.6 (78.1–84.8)	397	74.8 (70.8–78.4)	<0.001
Typical antipsychotics	783	42.7 (40.4–45.0)	558	45.9 (43.1–48.7)	196	34.9 (31.0–39.0)	141	26.6 (22.8–30.5)	<0.0001
Atypical antipsychotics	1146	62.5 (60.2–64.7)	770	63.3 (60.5–66.0)	343	61.1 (57.0–65.2)	309	58.2 (53.9–62.4)	0.220
Antidepressants	590	32.2 (30.0–34.4)	386	31.7 (29.1–34.4)	181	32.3 (28.4–36.3)	170	32.0 (28.1–36.2)	0.923
Mood stabilizers	280	15.3 (13.7–17.0)	190	15.6 (13.6–17.8)	81	14.4 (11.6–17.6)	90	17.0 (13.9–20.4)	0.116
ADHD medication	122	6.7 (5.6–7.9)	87	7.2 (5.8–8.8)	27	4.8 (3.2–6.9)	37	7.0 (5.0–9.5)	0.023
Hypnotics and sedatives	693	37.9 (35.6–40.1)	450	37.0 (34.9–39.8)	210	37.4 (33.4–41.6)	149	28.1 (24.3–32.1)	<0.001
Antiepileptics	124	6.8 (5.7–8.0)	85	7.0 (5.6–8.6)	34	6.1 (4.2–8.4)	34	6.4 (4.5–8.8)	0.683
Opioids	21	1.2 (0.7–1.8)	12	1.0 (0.5–1.7)	8	1.4 (0.6–2.8)	11	2.1 (1.0–3.7)	0.248
Drugs for addictive disorders	117	6.4 (5.3–7.6)	84	6.9 (5.6–8.5)	28	5.0 (3.3–7.1)	36	6.8 (4.8–9.3)	0.105
Psychotropic polypharmacy all	1295	70.6 (68.5–72.7)	870	71.5 (68.9–74.1)	373	66.5 (62.4–70.4)	321	(56.2–64.6)	0.014
2 psychotropic medications	449	24.5 (22.5–26.5)	280	23.0 (20.7–25.5)	152	27.1 (23.5–31.0)	144	27.1 (23.4–31.1)	0.813
3 psychotropic medications	372	10.3 (18.5–22.2)	248	20.4 (18.2–22.8)	112	20.0 (16.7–23.5)	91	17.1 (14.0–20.6)	0.340
4 psychotropic medications	243	13.3 (11.7–14.9)	173	14.2 (12.3–16.2)	62	11.1 (8.6–13.9)	50	9.4 (7.1–12.2)	0.311
>=5 psychotropic medications	231	12.6 (11.1–14.2)	169	13.9 (12.0–16.0)	47	8.4 (6.2–11.0)	36	6.8 (4.8–9.3)	0.345

a Comparison of medication use at admission and discharge among individuals discharged before 2020. McNemar test for paired nominal data; only individuals with no missing medication information at both time-points (n = 495) are contributing to test-statistic; significance level = 0.001.

When comparing medication use at admission and discharge in the subgroup of discharged patients, we found that the use of antipsychotics significantly decreased (81.6–74.8%; p < 0.001), which was especially driven by a reduction in typical antipsychotics (34.9–26.6%; p < 0.0001). Further, hypnotics and sedative use decreased (37.4–28.1%; p > 0.001). Benzodiazepine use accounted for approximately 20% of the prescriptions in the class of hypnotics and sedatives and decreased similarly (admission: 7.3%, 95%CI: 5.3–9.8; discharge: 5.3%, 95%CI: 3.5–7.5), however, not significantly (p = 0.101). Most psychotropic classes were relatively stable, comparing admission and discharge (Table 3). This stability was also observed throughout the first four follow-ups after admission among patients with the respective number of follow-ups (eFigures 3 and 4).

**Figure 1.**
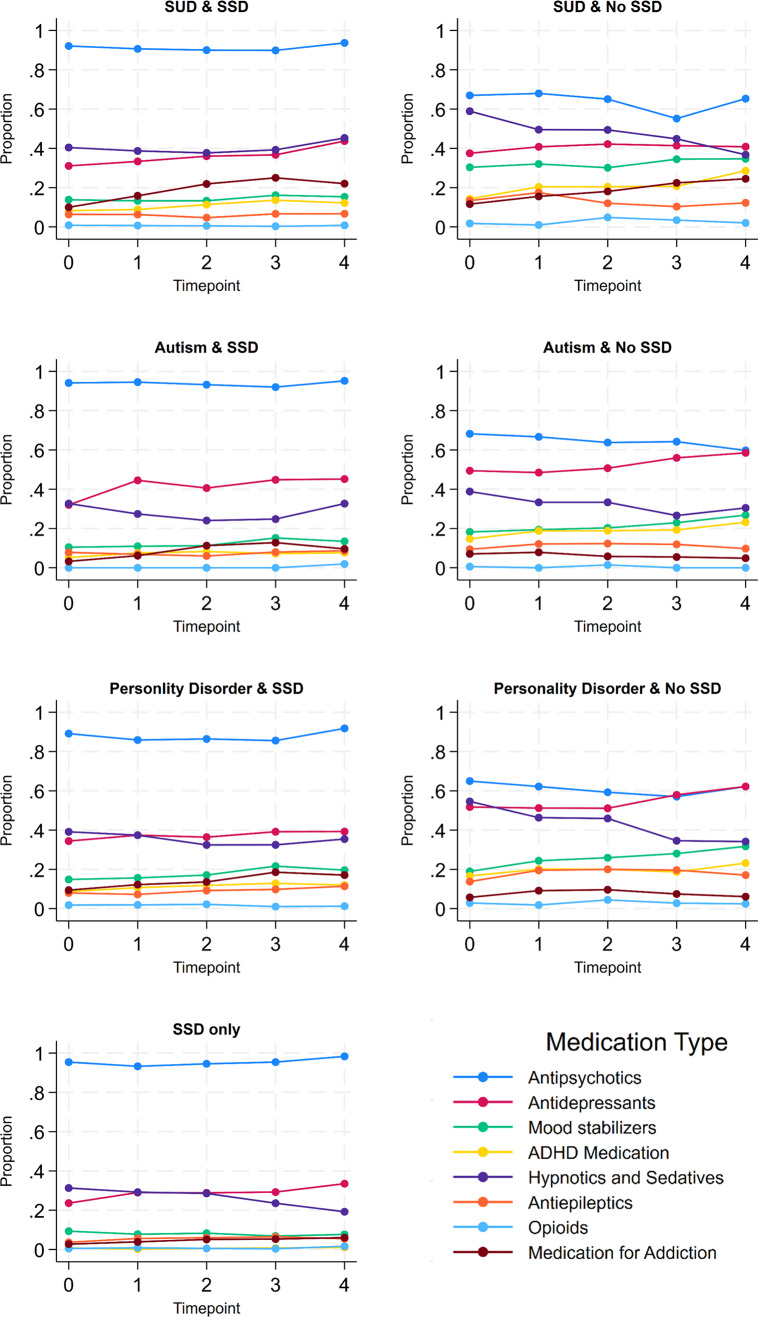
Use of antipsychotics and other psychotropic medication during the first four follow-ups after admission by diagnosis combinations (N at time point 0 = 1834; N at time point 4 = 707). SSD, Schizophrenia spectrum disorder; SUD, substance use disorder; ADHD, attention-deficit/hyperactivity disorder.

In the first 4 years of follow-up after admission, the use of antipsychotics was relatively stable in patients with SSD. The prevalence was approximately 90% across the follow-up and in every comorbidity combination (Figure 1 and eFigure 5). In patients without SSD, antipsychotics were the most used major psychotropic medication class, and the percentage of users was around 60% at any of the investigated time points (Figure 1). Comparing patients with and without SSD, medication use other than antipsychotics was more common among patients without SSD. This pattern was also observed in patients who had similar comorbidities besides SSD, e.g., ADHD medication was higher in those with autism and no SSD, compared to those with autism and SSD (Figure 1). The previously described decrease in the use of hypnotics and sedatives between admission and discharge (Table 3), is also visible within the first four follow-ups after admission in patients without SSD, and to some degree also in the group of patients with only SSD (Figure 1).

### Psychotropic polypharmacy

The prevalence of psychiatric polypharmacy was 70.6% (95%CI: 68.5–72.7) at admission. No significant differences at admission between sexes (eTable 2A) and subgroups for discharge status (eTable 2B) were detectable, except for the group of patients receiving five or more psychotropic medications simultaneously (eTable 2B). The proportion of patients receiving two or more psychotropic medications simultaneously per follow-up stayed stable within the first follow-ups and increased lightly toward the fourth follow-up (eFigure 6). However, following the group of discharged patients through time in care shows no significant change from admission to discharge (Table 3). The highest polypharmacy levels at admission were found in patients with ADHD, bipolar disorder, or recurrent depressive disorder (~85%) (eFigure 6A), and the lowest in those with only SSD (~65%) (eFigure 6B).

### Transitions of antipsychotic use

**Figure 2.**
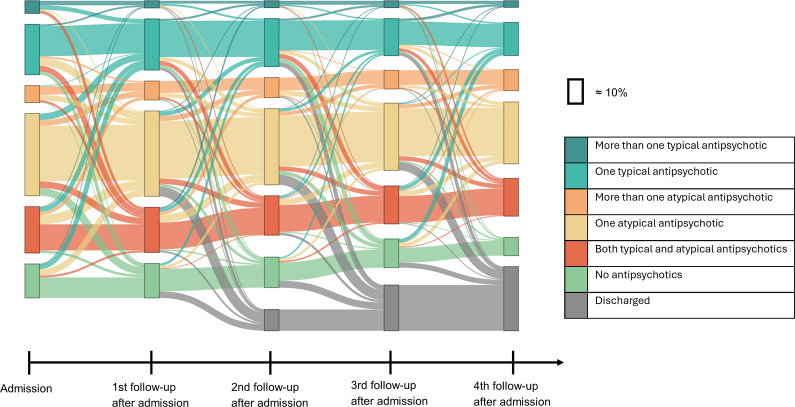
Use of antipsychotics in the first 4 years after admission. *Note*: Sample of patients that got admitted to forensic psychiatric care before 2016 and have no missing medication information (n = 843).

Figure 2 shows the number and types of antipsychotics that patients received at each follow-up from admission until discharge or until the fourth follow-up after admission, among patients who were admitted to FPC before 2016 and had no missing medication information at any of the presented time points (n = 843). In this sample, receiving one atypical antipsychotic was the most common treatment regime at every time point (33.9% at admission; and 34.7% of non-discharged patients at fourth follow-up). Overall, a few patterns were observed. From admission to the first follow-up, 40.0% of patients changed between treatment groups, with a majority leaving the group of one atypical antipsychotic or changing to it. Toward the second, third, and fourth follow-ups, fewer patients were changing the treatment regimes. Among patients not discharged at the respective time points, 28.6%, 27.2%, and 26.5% switched between treatment groups, respectively. Patients who received multiple typical antipsychotics at admission were the most likely to change treatment, with 63.6% changing into a different treatment group toward the first follow-up. A subgroup analysis comparing patients with and without discharge during the first four follow-ups is presented in eFigure 7 in the Supplementary Material.

## Discussion

This study examined the treatment patterns of psychotropic medications in FPC by estimating the prevalence of major medication classes and polypharmacy during care. We found a high prevalence of antipsychotic use in individuals with and without psychotic disorders, which remained at a similar level throughout the first years of care after admission. The prevalence of most other psychotropic medications was stable from admission to discharge, while the use of typical antipsychotics, as well as hypnotics and sedatives, declined. Transitions between different forms of antipsychotic use were common in the earlier stage of care. Psychotropic polypharmacy, as well as off-label antipsychotic use, was common throughout care in the observed time frame.

While there is no previous longitudinal study of psychotropic medication use in FPC, our findings of a high prevalence of antipsychotic use are similar to those reported in cross-sectional studies on compulsory psychiatric care in Cyprus and in a psychiatric inpatient ward in Portugal [19, 20]. This was expected, as antipsychotics are the main treatment for patients with psychotic symptoms and show good evidence in reducing relapse and violent crime [21, 22]. The observed use of around 90–95% in patients with SSD is, however, higher than in the general population of patients with psychotic disorders in Scandinavia, where a prevalence of 66–78% was found previously [11, 12]. A higher rate of antipsychotic use in FPC and other forms of inpatient care is likely driven by the severity of the conditions and the reduced possibility of declining treatment. Conversely, we observed in patients without SSD, antipsychotic use of around 60%. These numbers remained at a similar level, also after removing patients with bipolar disorders, which might indicate an off-label use of antipsychotics in patients without psychotic disorders. Off-label use of antipsychotics is common in general psychiatric care and used especially for personality disorder, anxiety, insomnia and aggressive behavior [23, 24]. Previous systematic reviews have shown that there is some evidence for the use of quetiapine in the treatment of generalized anxiety disorder [25] and a modest effect size in the reduction in aggressive behavior in those with psychotic disorders or dementia [26]. Further, there is literature suggesting that the use of antipsychotics in the treatment of personality disorder is associated with a reduction of violent criminality and suicidal behavior [27]. The lack of evidence for patients without these conditions and the known adverse effects suggest, nevertheless, that long-term off-label use of antipsychotics warrants further evaluation.

Antipsychotic polypharmacy was common in our sample. Around 30% of patients received multiple antipsychotics simultaneously at admission. Similar or higher rates of antipsychotic polypharmacy can also be found in forensic and non-forensic psychiatric settings elsewhere [9, 19, 20, 28]. There is, however, a lack of high-quality evidence on the effectiveness and safety of antipsychotic polypharmacy. Previous systematic reviews and meta-analyses on this topic show mixed results [29, 30]. The use of antipsychotic polypharmacy in FPC thus requires stronger evidence regarding effectiveness and safety.

Transitions between different forms of antipsychotic use, particularly in the earlier stage of the treatment, were notable. Antipsychotic treatment changes happened more often between admission and the first follow-up, compared to later time points. This shows that the initial period of care may involve a critical period of treatment optimization. Furthermore, most changes after admission are observed in those who initially are receiving multiple antipsychotics. This group might require more adjustment to achieve stabilization. The relative stability in subsequent follow-ups suggests that once an effective treatment is established, patients tend to maintain their regimen with fewer alterations. It remains unclear if this stability is driven by early optimization of the treatment, a lack of treatment re-evaluation and adaptation to adverse effects, or the lack of other treatment options to address the needs of the population. Further, due to the lack of dosage information, it is not possible to assess how the dosage of different antipsychotics is adapted throughout care. Nevertheless, our results underscore the importance of the initial treatment decisions for long-term treatment outcomes.

Psychiatric polypharmacy prevalence was observed to be stable at around 70% in the total sample throughout the first few years after admission to FPC, with an increase of around 4% toward the fourth follow-up. Among those discharged within the study period, slightly lower polypharmacy rates at admission compared to the rest of the sample were observed, but with no significant changes from admission to discharge. It could indicate that medication differences between patients with different lengths of stay in FPC are present already at admission. Patients with lower use of psychiatric polypharmacy might be discharged earlier, leading to a relative increase in polypharmacy toward the fourth follow-up. Looking at the use of psychotropic medication individually showed no substantial change within care, except for a decline in the use of typical antipsychotics and hypnotics and sedatives. Compared with general psychiatric patients with psychotic disorders, the prevalence of hypnotics and sedatives used at admission to FPC was higher (37.9% in FPC; 28% in general psychiatric patients with psychotic disorders) [12]. At discharge, however, the prevalence was more similar between those groups. Newly admitted patients to FPC are likely to experience heightened stress due to the committed crime, the legal process, the change of environment, and, in some cases, the withdrawal symptoms from the reduction of substance use. As a result, the need for sleep medication and treatment for anxiety is likely more pronounced in these patients than in general psychiatric patients with severe mental disorders. This holds especially for the first months of FPC and would also explain the reduction of hypnotics and sedatives use over time and the approximation to the prevalence of those outside FPC.

The major strength of this study is the globally unique data source, the nationwide FPC register. It allows an understanding of treatment choices within FPC based on a large and representative sample. Further, we chose a mixture of approaches to describe and visualize the treatment trends, contributing to a better understanding of current treatment strategies from different angles. There are, however, also several limitations. Firstly, we only had access to information on the medications recorded at each follow-up. Medications added and discontinued between the two follow-ups were not captured. However, since our results are characterized by a rather stable use for most medication classes throughout care, fluctuations between follow-ups can likewise be assumed to be of minor extent. Second, we lack information on dosages, lengths, and indications of the prescriptions, limiting our ability to further analyze the treatment patterns. Additionally, we are missing information on pro re nata prescriptions, except those which were given on more than three occasions in the week prior to a follow-up. This may cause the extent of polypharmacy to be underestimated. Third, the average missingness of medication information at an observation point is 6.5% and is not distinguishable from observations with no medication use (eMethod 1). Excluding those with missing data will lead to a small overestimation of the prevalence of psychotropic medication use.

Fourth, there were noticeable differences between patients who were discharged during the study period, compared to those who were still in care at the end of the study period. Patients with discharge information had a shorter mean stay and were more often in FPC without Special Court Supervision [15]. We acknowledged this by creating and comparing subgroups based on discharge information availability, to avoid generalizations from those with discharge information to the entire sample. Lastly, we only had access to up to three diagnoses per person per data entry as recorded in SNFPR, which could have caused some comorbidities of patients to be missed. However, as we focused only on diagnoses that are not characterized by an episodic nature and have a certain severity, this potential misclassification was likely affecting the results only to a small degree.

To conclude, this study identified a high prevalence of antipsychotic use, psychotropic polypharmacy, and off-label antipsychotic use throughout care in FPC in Sweden. The use of antipsychotics and most psychotropic medications remained relatively stable throughout time in care, except for a decrease in the use of typical antipsychotics, as well as hypnotics and sedatives. The study gives an overview of current pharmacological treatment and is an important contribution to the empirical understanding of FPC in Sweden, which is needed for evidence-based care planning and policymaking [13]. Foremost, this study provides an important knowledge base for the design of future studies, which are required to address critical questions regarding the effectiveness of different pharmacological treatment strategies in improving the well-being of patients and reducing their subsequent criminal behavior. SNFPR has large potential as a data source for future studies on medication use in FPC, especially once a wider spectrum of patients can be followed for their entire care period. A validation study of the medication information in SNFPR is, however, recommended to reduce the uncertainty introduced by the described limitations.

## Supporting information

10.1192/j.eurpsy.2025.10031.sm001 Sitter et al. supplementary material Sitter et al. supplementary material

## Data Availability

The Public Access to Information and Secrecy Act in Sweden prohibits individual-level data from being publicly shared. Researchers who are interested in replicating this study can apply for the data through the register holder (Registercentrum Västra Götaland: https://rattspsyk.registercentrum.se/).

## Supplementary material

The supplementary material for this article can be found at http://doi.org/10.1192/j.eurpsy.2025.10031.
