# Supplementary material for: Pharmacological treatment and psychiatric polypharmacy in forensic psychiatric care in Sweden
Source: Eur Psychiatry. 2025 Jun 11;68(1):e78. doi: 10.1192/j.eurpsy.2025.10031 (PMC12188328; doi:10.1192/j.eurpsy.2025.10031)
Supplement: Sitter et al. supplementary material [file S092493382510031Xsup001.docx]

**Supplements**

Contents

[eMethod 1. Missingness of medication information 2](#_Toc191978646)

[eMethod 2. Description of data records 2](#_Toc191978647)

[eMethod 3. Bonferroni correction of significance level 2](#_Toc191978648)

[eFigure 1. Flowchart of sample selection 3](#_Toc191978649)

[eFigure 2. Combinations of chronic psychiatric disease 4](#_Toc191978650)

[eFigure 3 5](#_Toc191978651)

[**A**. Number of patients in care during first four follow-ups after admission 5](#_Toc191978652)

[**B.** Number of patients in care with medication information during first the four follow-ups after admission 5](#_Toc191978653)

[eFigure 5. Use of antipsychotics and other psychotropic medication during the first 4 follow-ups after admission by diagnosis 7](#_Toc191978654)

[eFigure 6. Psychotropic Polypharmacy medication during the first 4 follow-ups after admission by diagnoses and diagnoses combinations 8](#_Toc191978655)

[eFigure 7. Use of Antipsychotics at admission and end of care or 4^th^ follow-up 9](#_Toc191978656)

[eTable 1. Categorization of Antipsychotics 10](#_Toc191978657)

[eTable 2 11](#_Toc191978658)

[**A.** Use of major medication classes at admission by sex 11](#_Toc191978659)

[**B.** Use of major medication classes at admission by discharge status 12](#_Toc191978660)

# eMethod 1. Missingness of medication information

In the data extraction of SNFPR, that we had access to, missing medication information is not sufficiently distinguishable from zero medication (no prescription of any psychotropic or any other form of medication), because in both cases it results in empty medication entries. We considered empty medication entries as missing for our analysis, due to the following reason: Based on previous reports, clinical studies and clinical experience, it was expected that around 2 – 3 % of patients in FPC in Sweden are not receiving psychotropic medication. This number is coherent with the number of patients in the study sample among those patients with no empty medication information (e.g. 2.8% at admission). The mean proportion of patients with empty medication information per time point is 6.5%. If we were to consider all those empty medication observations as zero medication, the proportion of patients without psychotropic medication would be substantially higher than what we would have expected from this setting, and therefore, it likely underestimates the amount of medication used in FPC.

- *Swedish National Forensic Psychiatric Register, RättspsyK (2022). Annual 2022. Gothenburg: Swedish National Forensic Psychiatric Register.*
  - *Bergman H MSc, PT, Nilsson T PhD, Psy, Andiné P PhD, MD, Degl'Innocenti A PhD, Psy, Thomeé R PhD, PT, Gutke A PhD, PT. Physical performance and physical activity of patients under compulsory forensic psychiatric inpatient care. Physiother Theory Pract. 2020 Apr;36(4):507-515.*

For all analyses, except for sample descriptions, only patients with non-missing medication information were included. If an individual had missing medication information at one time point, they were excluded from the analysis of the respective time point but still contributed to the analysis of other time points if those were non-missing.

# eMethod 2. Description of data records

Data records took place at admission, yearly follow-up, transfers between clinics, and discharge. A person who is in care for 4 years would typically have 5 records, with most of them being approximately one year apart. In some cases, records took place on the same day or within a few days. To maintain comparability with the rest of the sample, we excluded the earlier of two data records created within 10 days, assuming the latter one better reflects the subsequent period. An exception was made for entries showing a patient's admission, where subsequent records within less than 10 days were excluded.

# eMethod 3. Bonferroni correction of significance level

Bonferroni correction was used to adjust the significance level to account for an increased chance of Type 1 error due to multiple testing. In total 48 tests were performed, leading to a Bonferroni-adjusted significance level of 0.05/48 = 0.001.

# eFigure 1. Flowchart of sample selection

*
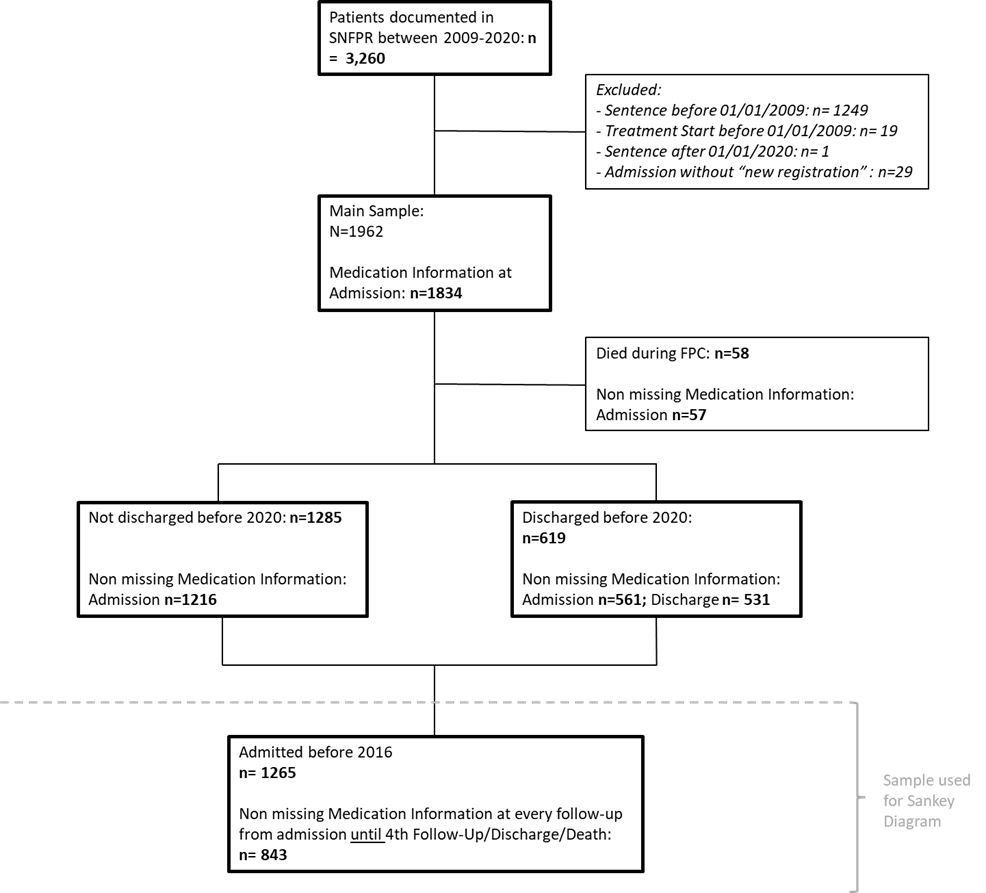
*

# eFigure 2. Combinations of chronic psychiatric disease

Upset-plot of the frequency of combinations of chronic psychiatric disease in the entire sample. The horizontal bars show the total number of patients with that specific diagnosis in the sample, while the vertical bars represent the count of patients that have specific diagnosis combinations. The dots and lines at the bottom visualise each intersection set. Only the 30 most common diagnosis intersections are represented in this graphic.


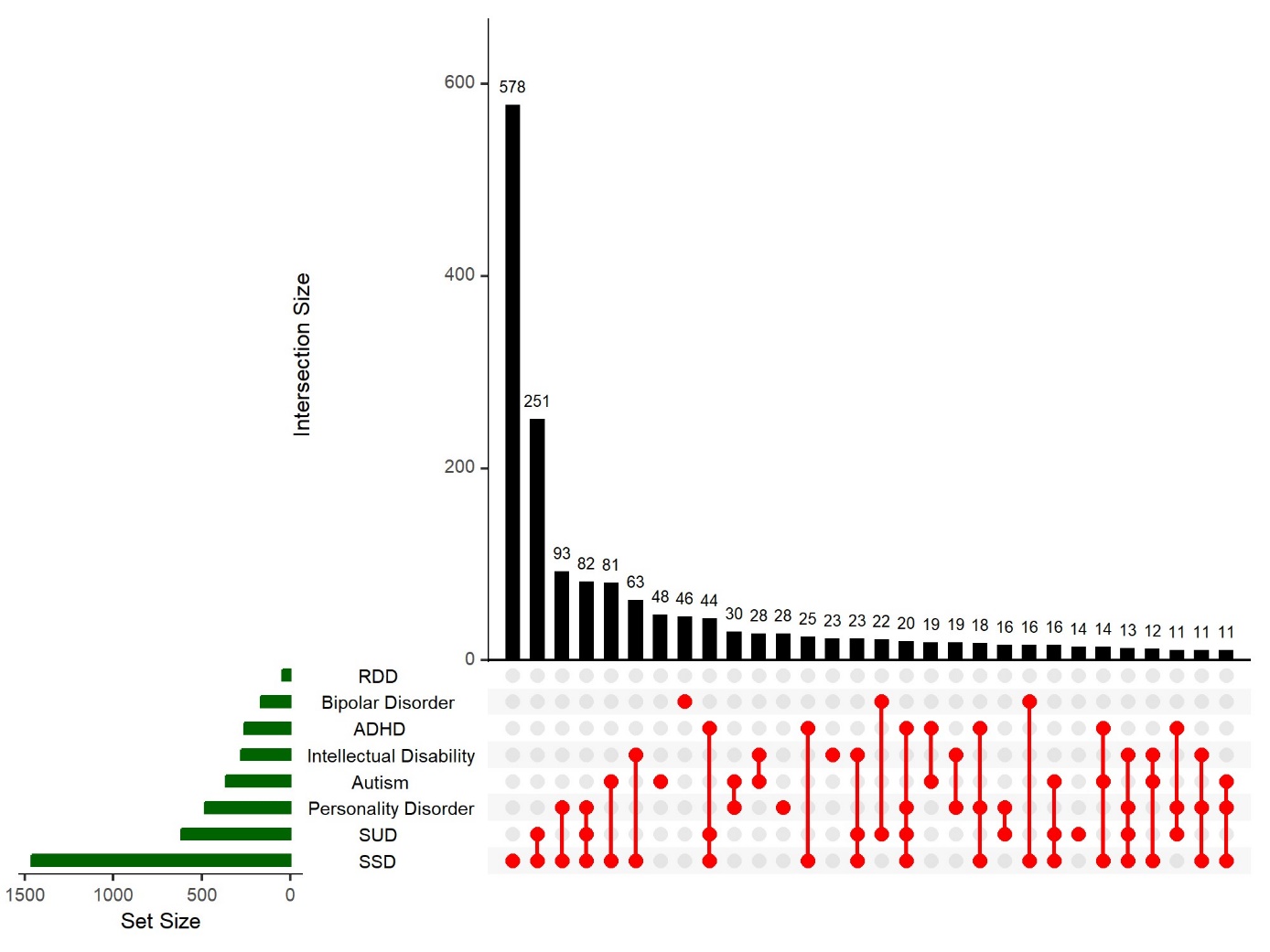


Note. SSD= Schizophrenia Spectrum Disorder, SUD= Substance Use Disorder, RDD=Recurrent Depressive Disorder, ADHD=Attention-deficit/hyperactivity disorder

# eFigure 3

## **A**. Number of patients in care during first four follow-ups after admission

**
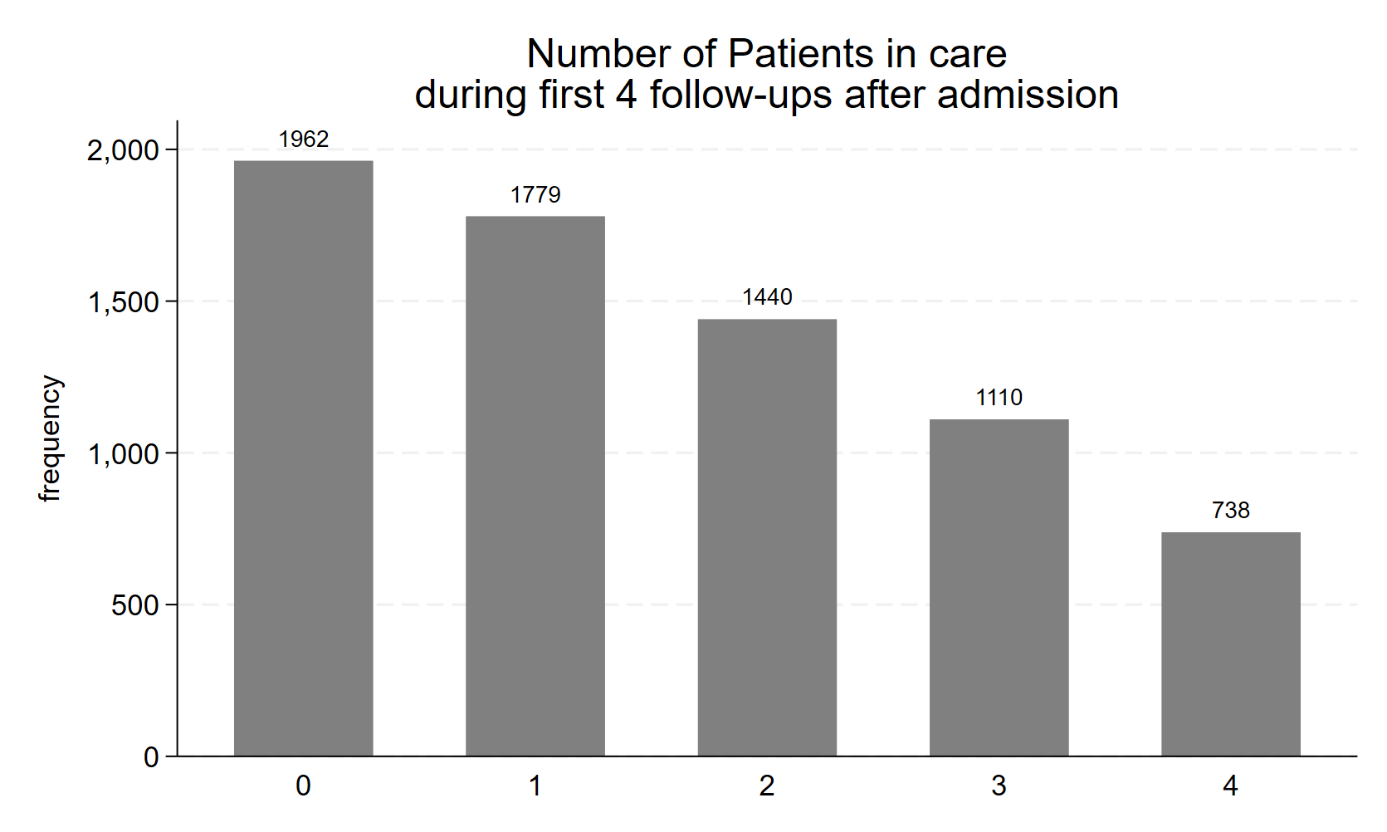
**

## **B.** Number of patients in care with medication information during first the four follow-ups after admission

**
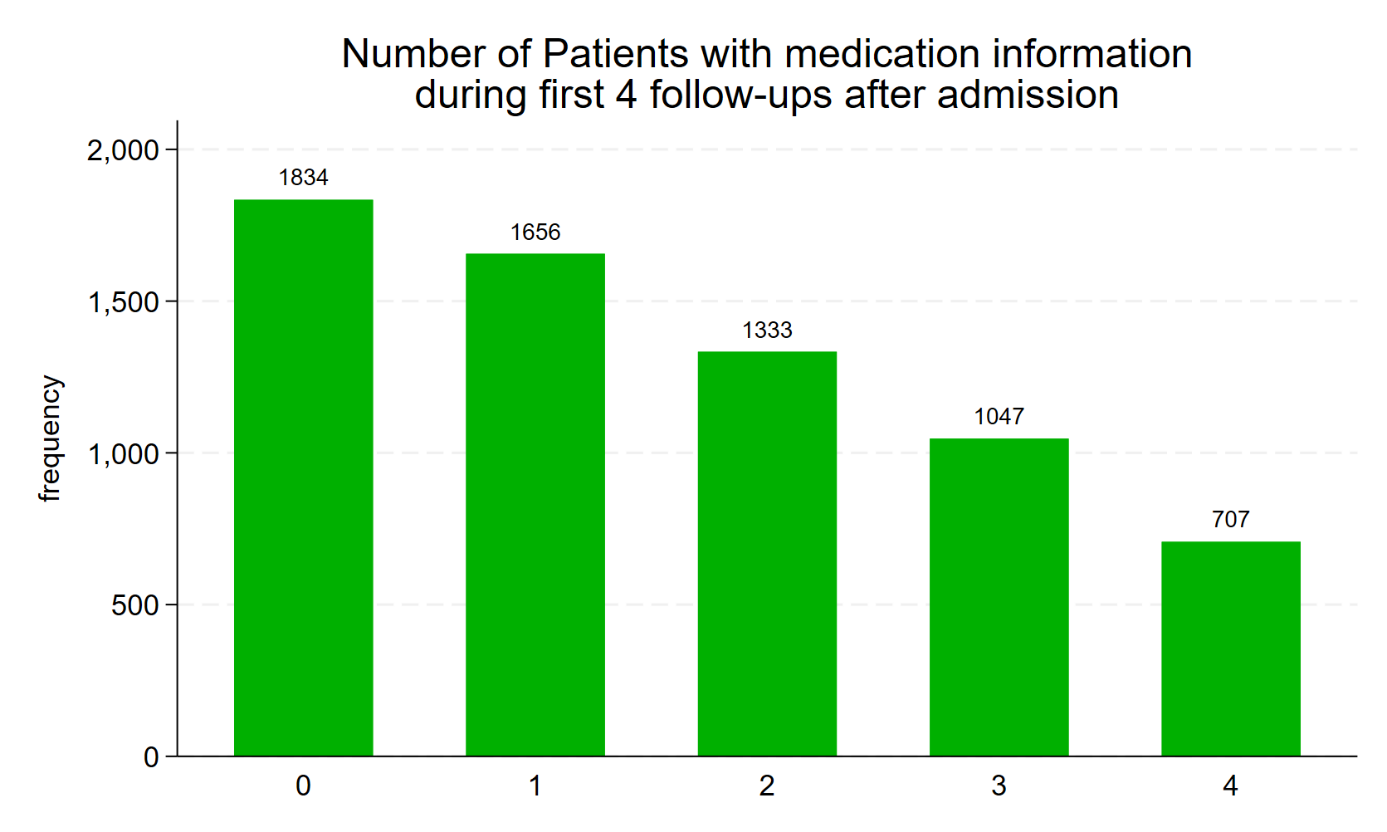
**

e**Figure 4**. Use of antipsychotics and other psychotropic medication during the first 4 follow-ups after admission

(N at timepoint 0 = 1834; N at timepoint 4 = 707)


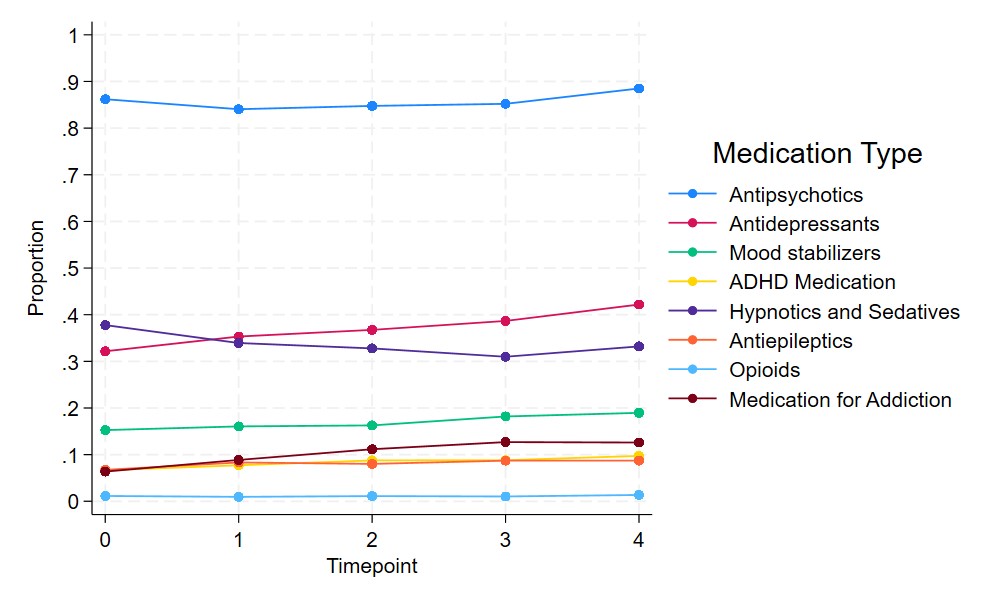


# eFigure 5. Use of antipsychotics and other psychotropic medication during the first 4 follow-ups after admission by diagnosis


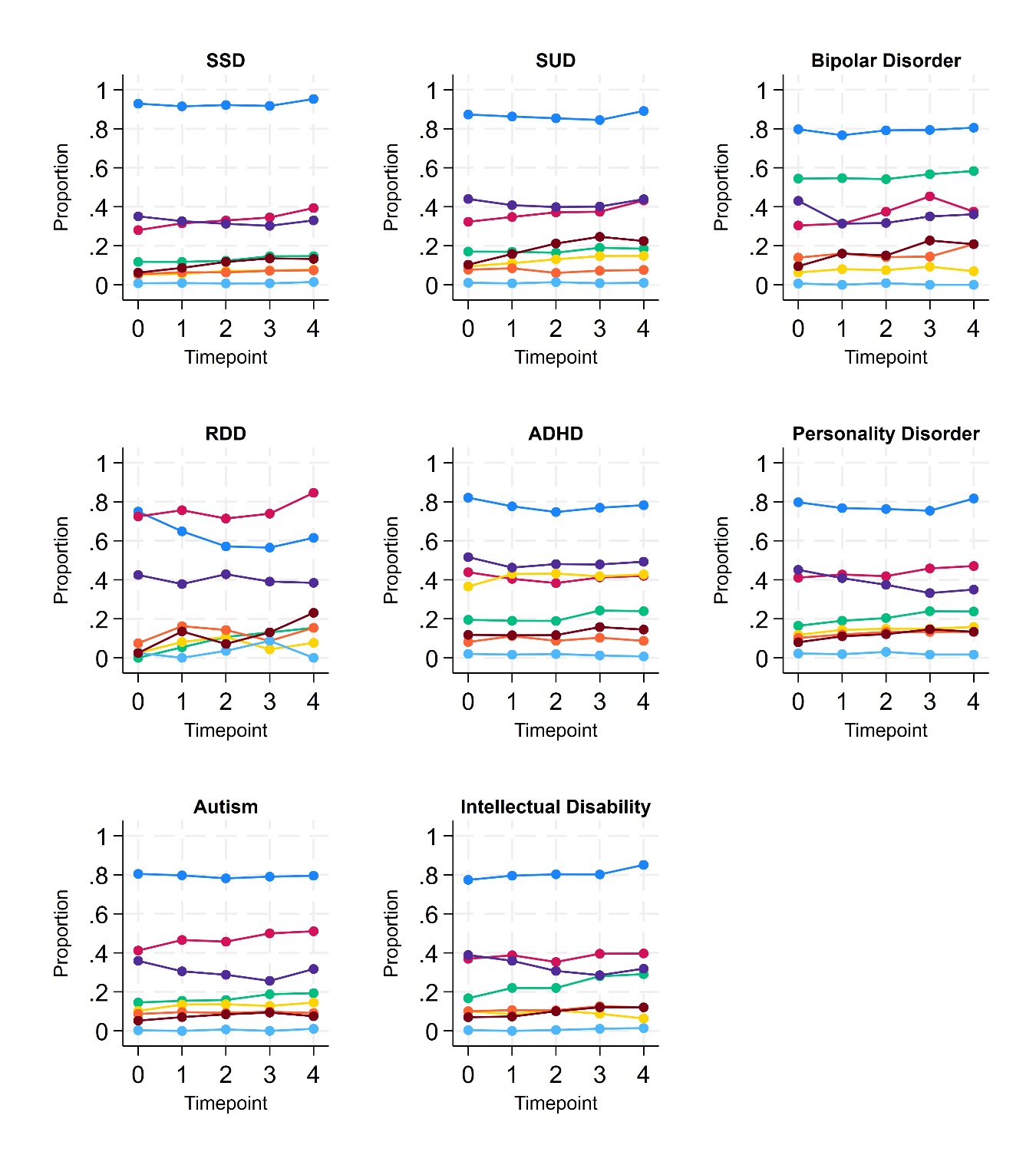

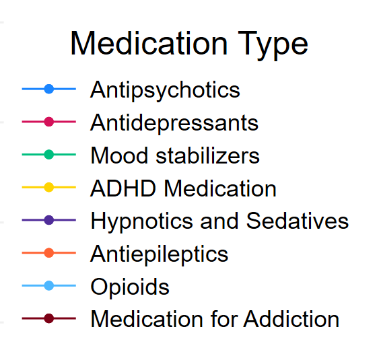


(N at timepoint 0 = 1834; N at timepoint 4 = 707)

Note. SSD= Schizophrenia Spectrum Disorder, SUD= Substance Use Disorder, RDD=Recurrent Depressive Disorder, ADHD=Attention-deficit/hyperactivity disorder

# eFigure 6. Psychotropic Polypharmacy medication during the first 4 follow-ups after admission by diagnoses and diagnoses combinations

(N at timepoint 0 = 1834; N at timepoint 4 = 707)

**A**


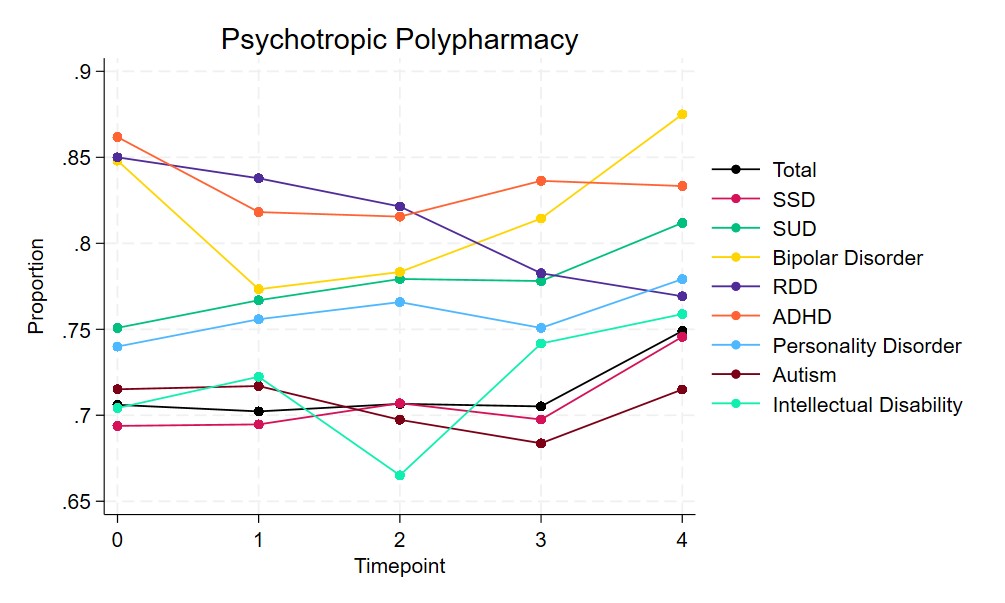


**B**


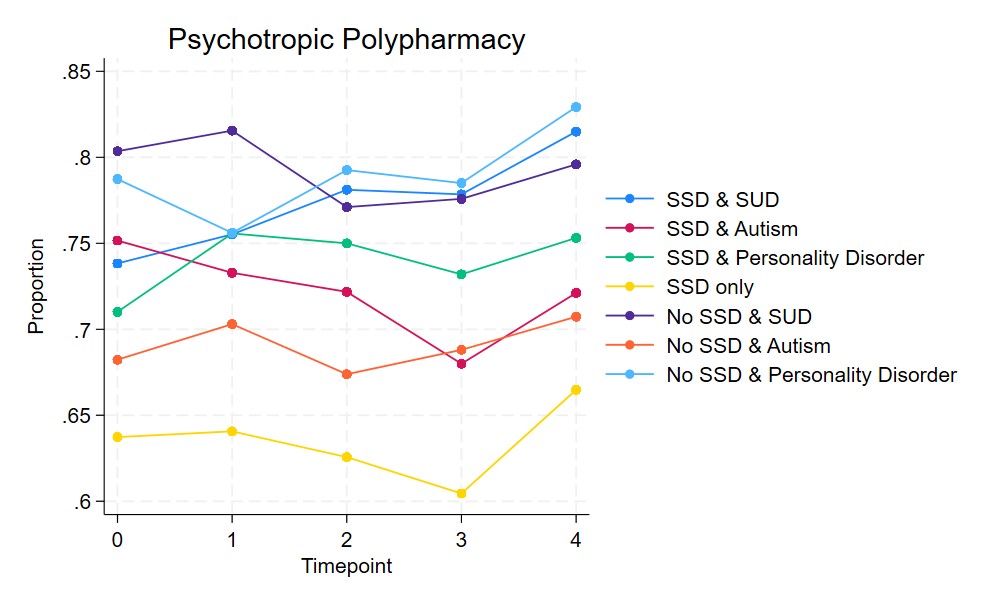
Note. SSD= Schizophrenia Spectrum Disorder, SUD= Substance Use Disorder, RDD=Recurrent Depressive Disorder, ADHD=Attention-deficit/hyperactivity disorder

# eFigure 7. Use of Antipsychotics at admission and end of care or 4^th^ follow-up


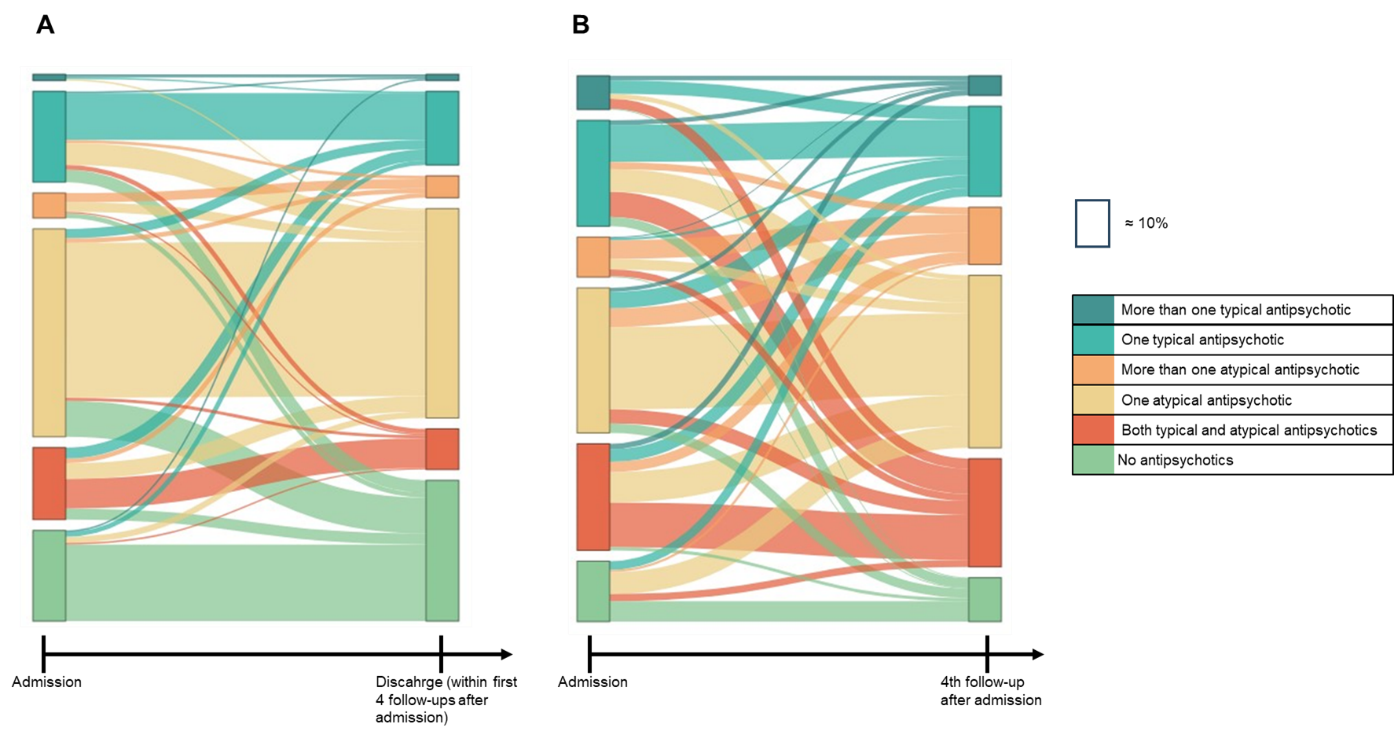


Note. Sample of patients admitted to care before 01/01/2016 and without missingness of medication information at admission and discharge/4^th^ follow-up (n=942) A: Patients, ending care within the first 4 follow-ups after admission (n=315); B: patients, not discharged yet within the first 4 follow-ups after admission (n=627)

# eTable 1. Categorization of Antipsychotics

| **Typical Antipsychotics** | **Atypical Antipsychotics** |
| --- | --- |
| N05AA01 | N05AL05 |
| N05AF03 | N05AX12 |
| N05AF01 | N05AX15 |
| N05AB02 | N05AH02 |
| N05AD01 | N05AE05 |
| N05AA02 | N05AD03 |
| N05AH01 | N05AH03 |
| N05AB03 | N05AX13 |
| N05AB04 | N05AH04 |
| N05AL01 | N05AX08 |
| N05AC02 | N05AE03 |
| N05AF05 | N05AL03 |
|  | N05AE04 |

# eTable 2

## **A.** Use of major medication classes at admission by sex

|  | **Admission** | | | | | |  |
| --- | --- | --- | --- | --- | --- | --- | --- |
| **Sample** (non-missing medication information) | **Total Sample (n=1834)** | | **Male (n=1505)** | | **Female (n=329)** | | **Comparison Male and Female** |
|  | **N** | **% (95% CI)** | **N** | **% (95% CI)** | **N** | **% (95% CI)** | **p-value*** |
| Antipsychotics | 1581 | 86.2 (84.5 – 87.8) | 1306 | 86.8 (85.0 – 88.4) | 275 | 83.6 (79.1 – 87.4) | 0.128 |
| Typical Antipsychotics | 783 | 42.7 (40.4 – 45.0) | 634 | 42.1 (39.6 – 44.7) | 149 | 45.3 (39.8 – 50.8) | 0.293 |
| Atypical Antipsychotics | 1146 | 62.5 (60.2 – 64.7) | 950 | 63.1 (60.6 – 65.6) | 196 | 59.6 (54.1 – 64.9) | 0.228 |
| Antidepressants | 590 | 32.2 (30.0 – 34.4) | 470 | 31.2 (28.9 – 33.6) | 120 | 36.5 (31.3 – 41.9) | 0.065 |
| Mood stabilizers | 280 | 15.3 (13.7 – 17.0) | 219 | 14.6 (12.8 – 16.4) | 61 | 18.5 (14.5 – 23.2) | 0.068 |
| ADHD Medication | 122 | 6.7 (5.6 – 7.9) | 99 | 6.6 (5.4 – 8.0) | 23 | 7.0 (4.5 – 10.3) | 0.785 |
| Hypnotics and sedatives | 693 | 37.9 (35.6 – 40.1) | 552 | 36.7 (34.2 – 39.2) | 141 | 42.9 (37.4 – 48.4) | 0.036 |
| Antiepileptics | 124 | 6.8 (5.7 – 8.0) | 83 | 5.5 (4.4 – 6.8) | 41 | 12.5 (9.1 – 16.5) | <0.001 |
| Opioids | 21 | 1.2 (0.7 – 1.8) | 15 | 1.0 (0.6 – 1.6) | 6 | 1.82 (0.7 – 3.9) | 0.202 |
| Drugs for addictive disorders | 117 | 6.4 (5.3 – 7.6) | 98 | 6.5 (5.3 – 7.9) | 19 | 5.8 (3.5 – 8.9) | 0.620 |
| Psychotropic Polypharmacy Total | 1295 | 70.6 (68.5 – 72.7) | 1048 | 69.6 (67.2 – 72.0) | 247 | - 1. (70.0 – 79.7) | 0.050 |
| 2 psychotropic medications | 449 | 24.5 (22.5 – 26.5) | 369 | 24.5 (22.4 – 26.8) | 80 | 24.3 (19.8 – 29.3) | 0.938 |
| 3 psychotropic medications | 372 | 10.3 (18.5 – 22.2) | 301 | 20.0 (18.0 – 22.1) | 71 | 21.6 (17.3 – 26.4) | 0.518 |
| 4 psychotropic medications | 243 | 13.3 (11.7 – 14.9) | 202 | 13.4 (11.7 – 15.2) | 41 | 12.5 (9.1 – 16.5) | 0.642 |
| >=5 psychotropic medications | 231 | 12.6 (11.1 – 14.2) | 176 | 11.7 (10.1 – 13.4) | 55 | 16.7 (12.8 – 21.2) | 0.013 |
| *Chi-squared test; significance level=0.001 | | | | | | | |

## **B.** Use of major medication classes at admission by discharge status

| **Time Point** | **At Admission** | | | | | | **Comparison individuals with and without discharge before 2020** |
| --- | --- | --- | --- | --- | --- | --- | --- |
| **Sample** (patients with non-missing medication information) | **Total Sample (n=1834)** | | **Individuals not discharged before 2020 (n= 1216)** | | **Individuals discharged before 2020 (n=561)** | |  |
|  | **N** | **% (95% CI)** | **N** | **% (95% CI)** | **N** | **% (95% CI)** | **p-value*** |
| Antipsychotics | 1581 | 86.2 (84.5 – 87.8) | 1072 | 88.6 (86.2 – 89.9) | 458 | 81.6 (78.1 – 84.8) | >0.001 |
| Typical Antipsychotics | 783 | 42.7 (40.4 – 45.0) | 558 | 45.9 (43.1 – 48.7) | 196 | 34.9 (31.0 – 39.0) | >0.001 |
| Atypical Antipsychotics | 1146 | 62.5 (60.2 – 64.7) | 770 | 63.3 (60.5 – 66.0) | 343 | 61.1 (57.0 – 65.2) | 0.377 |
| Antidepressants | 590 | 32.2 (30.0 – 34.4) | 386 | 31.7 (29.1 – 34.4) | 181 | 32.3 (28.4 – 36.3) | 0.827 |
| Mood stabilizers | 280 | 15.3 (13.7 – 17.0) | 190 | 15.6 (13.6 – 17.8) | 81 | 14.4 (11.6 – 17.6) | 0.518 |
| ADHD Medication | 122 | 6.7 (5.6 – 7.9) | 87 | 7.2 (5.8 – 8.8) | 27 | 4.8 (3.2 – 6.9) | 0.061 |
| Hypnotics and sedatives | 693 | 37.9 (35.6 – 40.1) | 450 | 37.0 (34.9 – 39.8) | 210 | 37.4 (33.4 – 41.6) | 0.863 |
| Antiepileptics | 124 | 6.8 (5.7 – 8.0) | 85 | 7.0 (5.6 – 8.6) | 34 | 6.1 (4.2 – 8.4) | 0.466 |
| Opioids | 21 | 1.2 (0.7 – 1.8) | 12 | 1.0 (0.5 – 1.7) | 8 | 1.4 (0.6 – 2.8) | 0.415 |
| Drugs for addictive disorders | 117 | 6.4 (5.3 – 7.6) | 84 | 6.9 (5.6 – 8.5) | 28 | 5.0 (3.3 – 7.1) | 0.122 |
| Psychotropic Polypharmacy All | 1295 | 70.6 (68.5 – 72.7) | 870 | 71.5 (68.9 – 74.1) | 373 | 66.5 (62.4 – 70.4) | 0.031 |
| 2 psychotropic medications | 449 | 24.5 (22.5 – 26.5) | 280 | 23.0 (20.7 – 25.5) | 152 | 27.1 (23.5 – 31.0) | 0.063 |
| 3 psychotropic medications | 372 | 10.3 (18.5 – 22.2) | 248 | 20.4 (18.2 – 22.8) | 112 | 20.0 (16.7 – 23.5) | 0.834 |
| 4 psychotropic medications | 243 | 13.3 (11.7 – 14.9) | 173 | 14.2 (12.3 – 16.2) | 62 | 11.1 (8.6 – 13.9) | 0.066 |
| >=5 psychotropic medications | 231 | 12.6 (11.1 – 14.2) | 169 | 13.9 (12.0 – 16.0) | 47 | 8.4 (6.2 – 11.0) | 0.001 |
| *Chi-squared test; significance level=0.001 | | | | | | | |
